# Etiology of Acute Rheumatism

**Published:** 1897-03

**Authors:** 


### ETIOLOGY OF ACUTE RHEUMATISM.

At the Hunterian Society, Dr. McClymont read a paper on this subject, in which he said that the factors at work were intrinsic and extrinsic. Evidences of a specific agent were obtained from various sources. As a rule the males were more subject than the females, but in his experience it was the reverse. The existence of a rheumatic diathesis was supported and inheritance was usually through the mother. The influence of lactic acid was discussed, but he could not entirely agree with those who advanced this. It was conceivable that by causing the formation of sarcolactic acid chills and colds predisposed the patient to the attack. Dr. Newsholme had shown that acute rheumatism was an urban disease and occurred more often in places situated in valleys; it occurred also when the mean soil-temperature was raised. He had also shown that where the soil-water was in excess acute rheumatism was very prevalent. The epidemic prevalence of the disease was fully discussed. "Rheumatic houses" were referred to and examples quoted, and the varying rheumatic manifestations occurring in these houses were strongly in favor of an infective cause. It was shown that the pathology of the disease was very similar to that of the group of diseases of infective character. Further, there was in acute rheumatism a selective character, especially as to age, as, for example, chorea in children and tonsilitis in adolescents. The presence of a specific virus had been recorded by various continental observers, but their observations were at variance. Dr. McClymont had made several observations, and after seven trials he obtained diplococci, and he promised further communications on the subject at a later date.—(*British Medical Journal.*)

